# Subject-Specific Cognitive Workload Classification Using EEG-Based Functional Connectivity and Deep Learning

**DOI:** 10.3390/s21206710

**Published:** 2021-10-09

**Authors:** Anmol Gupta, Gourav Siddhad, Vishal Pandey, Partha Pratim Roy, Byung-Gyu Kim

**Affiliations:** 1 Department of Computer Science and Engineering, Indian Institute of Technology, Roorkee 247667, India; agupta@cs.iitr.ac.in (A.G.); g_siddhad@cs.iitr.ac.in (G.S.); partha@cs.iitr.ac.in (P.P.R.); 2 Department of Biomedical Engineering, Institute of Nuclear Medicine and Allied Sciences, Defence Research and Development Organization, Delhi 110054, India; vishalp055@gmail.com; 3 Department of IT Engineering, Sookmyung Women’s University, Seoul 04310, Korea

**Keywords:** CNN, cognitive workload, functional connectivity analysis, LSTM, mental workload, mutual information, phase locking value, phase transfer entropy

## Abstract

Cognitive workload is a crucial factor in tasks involving dynamic decision-making and other real-time and high-risk situations. Neuroimaging techniques have long been used for estimating cognitive workload. Given the portability, cost-effectiveness and high time-resolution of EEG as compared to fMRI and other neuroimaging modalities, an efficient method of estimating an individual’s workload using EEG is of paramount importance. Multiple cognitive, psychiatric and behavioral phenotypes have already been known to be linked with “functional connectivity”, i.e., correlations between different brain regions. In this work, we explored the possibility of using different model-free functional connectivity metrics along with deep learning in order to efficiently classify the cognitive workload of the participants. To this end, 64-channel EEG data of 19 participants were collected while they were doing the traditional n-back task. These data (after pre-processing) were used to extract the functional connectivity features, namely Phase Transfer Entropy (PTE), Mutual Information (MI) and Phase Locking Value (PLV). These three were chosen to do a comprehensive comparison of directed and non-directed model-free functional connectivity metrics (allows faster computations). Using these features, three deep learning classifiers, namely CNN, LSTM and Conv-LSTM were used for classifying the cognitive workload as low (1-back), medium (2-back) or high (3-back). With the high inter-subject variability in EEG and cognitive workload and recent research highlighting that EEG-based functional connectivity metrics are subject-specific, subject-specific classifiers were used. Results show the state-of-the-art multi-class classification accuracy with the combination of MI with CNN at 80.87%, followed by the combination of PLV with CNN (at 75.88%) and MI with LSTM (at 71.87%). The highest subject specific performance was achieved by the combinations of PLV with Conv-LSTM, and PLV with CNN with an accuracy of 97.92%, followed by the combination of MI with CNN (at 95.83%) and MI with Conv-LSTM (at 93.75%). The results highlight the efficacy of the combination of EEG-based model-free functional connectivity metrics and deep learning in order to classify cognitive workload. The work can further be extended to explore the possibility of classifying cognitive workload in real-time, dynamic and complex real-world scenarios.

## 1. Introduction

Cognitive workload is the measure of the amount of mental effort required to complete any task [1]. Working memory is required to process information for short periods of time, while long-term memory is associated with storing information for long periods of time [2]. Tasks such as arithmetic operations, reading and learning require efficient use of working memory. Cognitive workload can be defined as the amount of mental activity utilized by working memory to complete any task. Assessment of an individual’s cognitive workload is an essential component in most human-machine collaboration tasks. A major application of this lies in the defense domain. Operations like driving under high-stress environmental conditions, monitoring air traffic control, piloting an aircraft or operating an unmanned vehicle are excellent examples. The optimal level of cognitive workload is pivotal in high-risk scenarios where important decisions are supposed to be made in real-time. The rate at which the information is processed determines the workload induced in any individual while performing any task. A high workload can lead to unplanned and disproportionate hazards, and too little workload can lead to being disengaged from the task. This points to the importance of maintaining optimal cognitive workload in high-risk scenarios to perform the task satisfactorily. With respect to cognitive workload, emotional intelligence and stability are regarded as essential components. An individuals’ cognitive load will be affected by emotional valence as it will interfere with parallel cognitive processing. Studies show a positive relation between emotional intelligence and some cognitive tasks [3,4]. Therefore, classification of cognitive workload can be an essential indicator of emotional intelligence and stability.

Although the assessment of cognitive workload is important, it is not a trivial task. Traditional methods of the evaluation of cognitive workload included subjective measures such as interviews or questionnaire-based approaches where the participants self-reported the amount of workload caused/induced during the task. Various research groups such as Hart et al. [5] and Malekpour et al. [6] contribute towards the assessment of cognitive workload with the use of subjective methods, primarily in the form of self-assessment questionnaires, like NASA-TLX (National Aeronautics and Space Administration Task Load Index), MCH (Modified Cooper-Harper Scale) and SWAT (Subjective Workload Assessment Test). Such questionnaires generally record the various metrics involved in performing the task, such as demand (mental, physical and temporal), effort, pressure, concentration, frustration, etc., to evaluate their connection with performance during the task. These methods prove to be subjective to the individual participant, however, and can be biased and prove to be unreliable as a distinct and coherent metric for the evaluation and estimation of cognitive workload in general as they depend on the participant recalling past engagement. Another drawback of using post-task questionnaire is that it does not allow for real-time evaluation of cognitive workload.

In contrast to the subjective questionnaire based methods, the evaluation based on neuro-physiological signals present an opportunity for an objective and real time assessment of cognitive workload. However, this method of evaluation comes at the expense of limited availability of equipment, trained operators and high costs. To obtain better efficacy and efficiency, physiological measures such as Electroencephalography(EEG), Event-Related Potential (ERP), Eye Tracking (gaze entropy), and Heart Rate Variability (HRV) can be utilized [7,8,9]. EEG is highly accepted as a measure to assess cognitive workload in real-time [10,11,12]. Various EEG features including time, frequency, time-frequency, and spatial domain features extracted from raw EEG data are effective ways to gain information from EEG signals. Time domain features mainly include Event Related Potentials (ERP) [13], statistical features (mean, standard deviation, variance, etc.), higher-order crossing analysis [14], and Hjorth parameter. Frequency domain features include decomposing the frequency in multiple sub-bands such as delta, theta, alpha, beta, and gamma bands which are mainly associated with deep sleep, drowsy, relaxed, engaged, conscious, and active states, respectively [15]. Such features are commonly used for classification of workload in various machine learning experiments. Recent advancements in the application of deep learning in various domains such as emotion recognition, pattern recognition and prediction makes it an excellent choice to be used with EEG signals for classification [16,17,18,19]. EEG signals can be used to decode and classify the human cognitive state. Various studies have carried out research in the area with different combinations of EEG features and machine learning models. Bashivan et al. [20] demonstrates the use of fast Fourier transform to convert EEG data into the frequency domain and map the 3D spatial positions of electrodes to 2D, according to the distribution of the electrodes. Using theta, alpha and beta frequency bands, 3-channel spectral maps are generated and sent to CNN model for classification of mental load. Kwak et al. [21] propose a multi-level feature fusion method based on CNN to learn the spectral, spatial, as well as local and global information. Li et al. [22] reviews some deep learning models (e.g., RNN and CNN) and their applications for EEG data to decode brain activities and diagnose brain diseases.

Substantial research for estimation of cognitive workload from EEG using machine learning and deep learning is limited. Most of the studies perform binary classification of workload into high and low by extracting compute expensive EEG features from the raw data, making these non ideal to be used in real life conditions or in real time. Das et al. [23] reports an accuracy of 86.33% and 82.57% for binary and three class classification, respectively, using a BLSTM-LSTM based architecture in a subject independent study. Appriou et al. [24] performs subject specific and subject independent studies for binary classification of workload, achieving the highest mean accuracy of 72.7% and 63.7% using CNN for subject-specific and subject independent cases, respectively. In the study by Zhang et al. [25], the authors achieved an accuracy of 88.9% in binary classification using a combination of RNN and 3D CNN models with EEG topographic maps as features for classification. Using a similar technique of topographic maps in combination with a modified CNN model, highest accuracy of 91.9% in subject specific three class classification is reported [26]. However, more informative features regarding an individual’s brain can be obtained from EEG data. Information acquired from signals originating from a specific brain region can be regarded to represent the brain activity of that region. This allows the study of separate brain regions in isolation when evaluating characteristics relevant to a specific cognitive state and this methodology has been adopted by various researchers. However, neuronal activity is not this straightforward as different regions of the brain contribute to the completion of a task, while different regions are still dominantly responsible for specific functions required for the completion of the task. This implores the necessity of examining the inter-regional interactions to understand the collaboration of the different brain regions. More formally, this analysis is termed as brain connectivity.

Brain Connectivity has been used to study the nature of the cerebrum in the past. Based on the attributes of connections, it can be classified into three types: structural connectivity (biophysical connections between neurons or neural elements), functional connectivity (statistical relations between anatomically un-connected cerebral regions) and effective connectivity (directional causal effects from one neural element to another) [27]. This study focuses on the exploration of functional brain connectivity as a measure to assess different levels of workload. Brain functional connectivity has been linked with cognitive deficient psycho-physiological diseases. Strong patters on connectivity in resting state EEG are evident in autism spectrum disorders as reported by [28]. Slower and less efficient connectivity is found in schizophrenia patients as reported by [29]. Another study suggested a relation between high frequency connectivity neural pattern and recurrent illness course of major depressive disorder [30]. However, few studies have investigated the links between cognitive workload and brain functional connectivity networks. Dimitrakopoulos et al. [31] is one such study that has used brain connectivity measure as a feature for classification of workload. This study uses correlation as a method of brain connectivity and achieved an accuracy of 88% for binary classification using SVM classifier. Another study by Islam et al. [32] explores the use of Mutual Information based functional connectivity for binary classification of drivers’ mental workload using the SVM classifier and obtained an accuracy of 82%. There are only a limited number of studies that explore functional connectivity as a feature for classification of workload. Therefore, in this study we explore different functional brain connectivity methods as features to be used for classification of levels of cognitive workload. EEG data is known to have high inter-subject variability [33,34]. Various researchers such as Byrne et al. [35] and Pang et al. [36] study the inter-subject variability. Nentwich et al. [37] report the subject-specific nature of EEG-based functional connectivity. Given this evidence, subject specific classification of workload has been aimed at in this study. In Zhang et al. [38], the authors compared the subject-dependent and independent approach and highlighted that variations in feature distribution of EEG across subjects reduces the generalization ability of a classifier and at the same time subject-dependent approach provides a promising way to solve the problem of personalized classification. In Neto et al. [39], the authors discussed various subject specific characteristics and data splitting techniques for EEG data. A possible advantage of subject specific classification is that the classifier can learn subject-dependent features and it can be really useful in building robust and effective BCI systems [40,41].

The contributions of this paper can be summarized as follows:

- A novel method of cognitive workload estimation using EEG, functional brain connectivity and deep learning is proposed. Our pipeline included cleaning 64-channel EEG data, selecting 16 electrodes based on brodmann area, extracting a 16 × 16 connectivity matrix and using deep neural networks for classifying workload into low, medium and high classes.
- We chose model-free functional connectivity metrics (Mutual Information (MI), Phase Lag Value (PLV) and Phase Transfer Entropy (PTE) to classify workload using simple yet effective deep learning architectures (CNN, LSTM and Conv-LSTM) in near real-time.
- The proposed method achieved state-of-the-art accuracy for three class workload classification. We achieved an average accuracy of 80.87% for three class workload classification problems using MI and CNN. PLV and PTE also perform better with CNN as compared to the other architectures with a average classification accuracy of 74.07% and 71.16%, respectively. CNN outperforms the other architectures because of the high spatial information in the input connectivity matrix.
- The efficacious results highlight the promise of using functional connectivity features of EEG for real-time workload classification.

The rest of the paper is organized as follows. Section 2 presents the materials and methods used for in the experiment. Section 3 discusses the results obtained in various experiments and Section 4 presents the implications of the reported results and the possible future directions and possible extensions of the current work.

## 2. Materials and Methods

### 2.1. Participants

A total of 19 participants (11 male and 8 females, mean age = 20.1 years, standard deviation = 1.2 years, minimum age = 19 years, maximum age = 23 years) at the Department of Biomedical Engineering, Institute of Nuclear Medicine and Allied Sciences, Delhi, India participated in this study. An institutional ethical committee approved the study at the Institute of Nuclear Medicine and Allied Sciences. Participation in the study was voluntary, and the subjects gave written consent before participating in the study. Out of 19 participants, 18 participants were right-handed, and one was left-handed. None of the participants reported neurological/psychological/mental history of any kind. All the participants hailed from a Science/Engineering/Technology/Mathematics (STEM) background. All the participants received a flat payment of INR 50, irrespective of their performance in the study.

### 2.2. The N-Back Task

**Figure 1:**
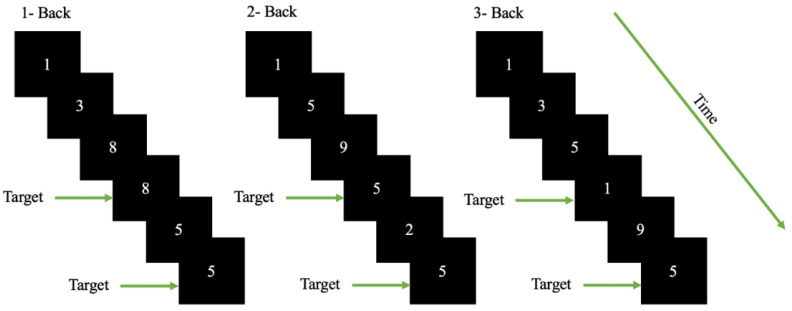
Schematic of the n-back task used for the cognitive workload classification. The participants were required to observe a sequence of single digits and determine whether the stimuli was a target. A target is the digit which is identical to the digit that appeared ’n’ digits back in the sequence. For example, in the 2-back scenario 5 is the target as the sequence of digits were 9,**5**,2,**5**.

The modern version of the n-back task [42] was designed using OpenSesame v 3.3.6 [43]. The n-back task is one of the most used psychological tests for inducing cognitive workload. In the task, the participants were required to observe a sequence of single digits separated by a small interval of time and for each letter they were required to identify whether the stimuli are a target (identical of the digit that has appeared ‘n’ digits back in the sequence) (see Figure 1). During a session/block the value of ‘n’ is kept constant. An increase in the value of ‘n’ induced cognitive workload according to [43]. The participants were required to interact with the appeared stimuli depending on the value of ‘n’.

A total of 339 sessions were presented to each participant in a randomized manner with 113 sessions each for 1, 2 and 3 back. The sessions were initialized with an instruction set that was displayed for 5 seconds, where the participants were informed about the nature of session (type of ‘n’). After the instruction block, the set of digits (1–9) appeared on the screen in sequence. The digits stayed on the screen for 500 ms, the participants were given 1500 ms to respond. The participants had to press space-bar in case the digit appeared was a target in accordance with the session. The inter-stimulus interval was 2000 ms (with 500 ms where the stimuli was displayed and 1500 ms given for response). The task was designed in accordance with standard n-back format. The n-back stimuli occurred within a visual angle of about 40° horizontally and about 4.50° vertically so the stimuli fall within the participants’ visual field and for minimal eye movement. The stimuli were presented using OpenSesame [43], an open-source experiment builder. The target missed was also considered as an incorrect response in this case. The first three session of each conditions (n-back) were removed from further data analysis.

### 2.3. Physiological Data Acquisition and Pre-Processing

Sixty-four channel EEG were recorded through Ag/AgCl electrodes conforming with the extended 10–20 electrode system of placement. An eego^TM^mylab amplifier (ANT Neuro, Enschede, The Netherlands) was used in the data acquisition. Electrooculogram (EOG) data was acquired from a single electrode placed below the right eye. All channels were grounded to channel CPz. Impedances were kept below 20 kΩ. The EEG data were sampled at 2048 Hz. The data were later downsampled to 256 Hz. During the recording process the participants were requested to sit in a relaxed posture to avoid potential contamination of data with movement artifacts. The data was referenced to linked mastoids in the further analyses. For pre-processing, DC offset was applied followed by band-pass with 0.1–45 Hz and finally we used ICA to get rid of the ocular and other artifacts. The data was then segmented according to the three conditions (1, 2 and 3 back) for all the 19 subjects.

### 2.4. Feature Extraction

Different cognitive tasks activate different specialized brain areas where the brain could dynamically coordinate the information flow to achieve the task [44]. Functional Connectivity is a method of quantifying these neuronal interactions. There exist many different algorithms for calculating these interactions using electrophysiological data. These algorithms can be divided into different domains based on the direction of the interaction among brain regions and interdependence of the signals [45]. In this study, we chose three connectivity metrics namely Mutual Information (MI), Phase Locking Value (PLV) and Phase Transfer Entropy (PTE). The reason for choosing these three metrics was to compare directed and non-directed model-free measures. One goal of the study was to build a near real-time framework for workload estimation using EEG, which is why only model-free connectivity measures were chosen. Therefore, we used only the raw (cleaned) EEG data to calculate the metrics.

Another important aspect for making the system fast was to select the dimensions of the connectivity matrix. To that end, 16 electrodes were chosen from the available 64. Choosing the 16 electrodes was done with brodmann areas in mind as functional connectivity implies interaction between different brain regions. In his article, Kaiser [46] defined a mapping between the EEG electrodes and different brodmann areas; therefore, we selected the same 16 EEG electrodes. The electrodes were Fp1, Fp2, F7, F3, F4, F8, T7, C3, C4, T8, P7, P3, P4, P8, O1 and O2. The closest associated brodmann areas with these electrodes are 10, 10, 47, 8, 8, 45, 42, 2, 1, 21, 37, 39, 39, 37, 18 and 18, respectively. This electrode placement is also supposed to be the most optimal for source localization [46]. We used the pre-processed EEG data to calculate these 16 × 16 functional connectivity metrics. Next, the different connectivity measures are discussed.

#### 2.4.1. Mutual Information (MI)

In information theory, MI is used to quantify the interdependence between two time series [47]. For a pair of discretized random variables *x* and *y* that are recorded from time series with their respective probability distribution functions P(x) and P(y), and joint probability function P(x,y), the MI between *x* and *y* can be defined as:

(1) $$MI_{xy}=\sum_{x\in X,y\in Y}P\left(x,y\right)\log\frac{P(x,y)}{P(x)P(y)}.$$

MI was proposed as a measure to quantify the strength of functional connectivity between a pair of time series data.

#### 2.4.2. Phase Locking Value (PLV)

Phase locking value (PLV) is a measure to quantify the synchronization of phase of different signals as acquired from separate brain areas. The analytical representations of two signals originating from brain regions, *k* and *l*, $s_{k}\left(t\right)$ and $s_{l}\left(t\right)$, are obtained by the Hilbert transform and expressed as [48,49]:

(2) $$z_{k}=A_{k}\left(t\right)e^{j\varphi k(t)},$$

(3) $$z_{l}=A_{l}\left(t\right)e^{j\varphi l(t)},$$

The differences in phase are then calculated at each time point by

(4) $$\Delta \varphi_{k,l}\left(t\right)=\varphi_{k}\left(t\right)-\varphi_{l}\left(t\right).$$

Thereafter, by averaging over all time points ($n_{t}$ being the number of time points) the PLV between the brain regions *k* and *l* is represented as:

(5) $$PLV\left(k,l\right)=\frac{1}{n_{t}}\left|\sum_{t=1}^{n_{t}}e^{j\Delta \varphi_{k,l}\left(t\right)}\right|,$$

The PLV ranges between 0 (which reflects no phase synchronization) and 1 (which reflects perfect phase synchronization). After the PLV calculation is repeated for all brain regions, it is assembled to form a connectivity matrix.

#### 2.4.3. Phase Transfer Entropy (PTE)

The flow of information between neuronal regions are quantified by the estimation of causal influence one region exercise on another. There is a plethora of methods to quantify the neuronal interactions, out of which PTE is the only measure that is phase-specific and directed in nature. For a connectivity metric to quantify the interactions amicably it should:

1. be robust to noise and linear mixing of signals [50,51]
2. computationally efficient
3. limit the number of a priori parameters
4. be able to detect transient frequency band from short data samples
5. allow the testing of statistical significance by constructing surrogate data from the experimental samples

PTE [52] is a method of quantifying directed phase interaction across trials as well as continuous data using binning methods for state-space reconstruction based on the same principle as Wiener-Granger causality [53]. In the framework of Information Theory, the Wiener-Granger causality can be re-written as: “a source signal has causal influence on the target signal, if the uncertainty of the target signal conditioned by the source signal and its own past is smaller than the uncertainty of the target signal conditioned by its own past” [54]. The instantaneous phase and amplitude of a signal *x*(*t*) can be expressed by its analytic associate as expressed in Equation (1). The PTE for an analysis lag θ can be defined as:

(6) $$PTE_{XY}=H\left(\varphi_{y}\left(t\right),\varphi_{y}\left(t^{\prime }\right)\right)+H\left(\varphi_{y}\left(t^{\prime }\right),\varphi_{x}\left(t^{\prime }\right)\right)-H\left(\varphi_{y}\left(t^{\prime }\right)\right)-H\left(\varphi_{y}\left(t\right),\varphi_{y}\left(t^{\prime }\right),\varphi_{x}\left(t^{\prime }\right)\right),$$

where $\varphi_{x}\left(t^{\prime }\right)$ and $\varphi_{y}\left(t^{\prime }\right)$ are the past states at lag θ, i.e., $\varphi_{x}\left(t^{\prime }\right)=\varphi_{x}\left(t-\theta \right)$ and $\varphi_{y}\left(t^{\prime }\right)=\varphi_{y}\left(t-\theta \right)$. The marginal and the joint entropies can then be defined as [55]:

(7) $$H\left(\varphi_{y}\left(t\right),\varphi_{y}\left(t^{\prime }\right)\right)=-\sum p\left(\varphi_{y}\left(t\right),\varphi_{y}\left(t^{\prime }\right)\right)\log p\left(\varphi_{y}\left(t\right),\varphi_{y}\left(t^{\prime }\right)\right),$$

(8) $$H\left(\varphi_{y}\left(t^{\prime }\right),\varphi_{x}\left(t^{\prime }\right)\right)=-\sum p\left(\varphi_{y}\left(t^{\prime }\right),\varphi_{x}\left(t^{\prime }\right)\right)\log p\left(\varphi_{y}\left(t^{\prime }\right),\varphi_{x}\left(t^{\prime }\right)\right),$$

(9) $$H\left(\varphi_{y}\left(t^{\prime }\right)\right)=-\sum p\left(\varphi_{y}\left(t^{\prime }\right)\right)\log p\left(\varphi_{y}\left(t^{\prime }\right)\right),$$

(10) $$H\left(\varphi_{y}\left(t\right),\varphi_{y}\left(t^{\prime }\right),\varphi_{x}\left(t^{\prime }\right)\right)=-\sum p\left(\varphi_{y}\left(t\right),\varphi_{y}\left(t^{\prime }\right),\varphi_{x}\left(t^{\prime }\right)\right)\log p\left(\varphi_{y}\left(t\right),\varphi_{y}\left(t^{\prime }\right),\varphi_{x}\left(t^{\prime }\right)\right),$$

where the probabilities are computed by histograms of occurrences of single, pairs or triplets of phase estimates in an epoch. The prediction delay θ and the number of bins in the histogram was set as $\left(\left(L\times CH\right)\right)⁄N_{\pm }$ and $e^{0.626+0.4\ln(L-\theta -1)}$ respectively, where *L* is the length of the epoch in sample count, CH is the number of channels and $N_{\pm }$ is the number of times the phase changed its sign across time and channels. The PTE values were normalized between 0 and 1 with $0.5<PTE_{xy}<0.5$ implying an information flow of x→y, $0<PTE_{xy}<0.5$ implying information flow preferentially of x←y and 0.5 implying no preferential flow of information.

### 2.5. Classification

**Figure 2:**
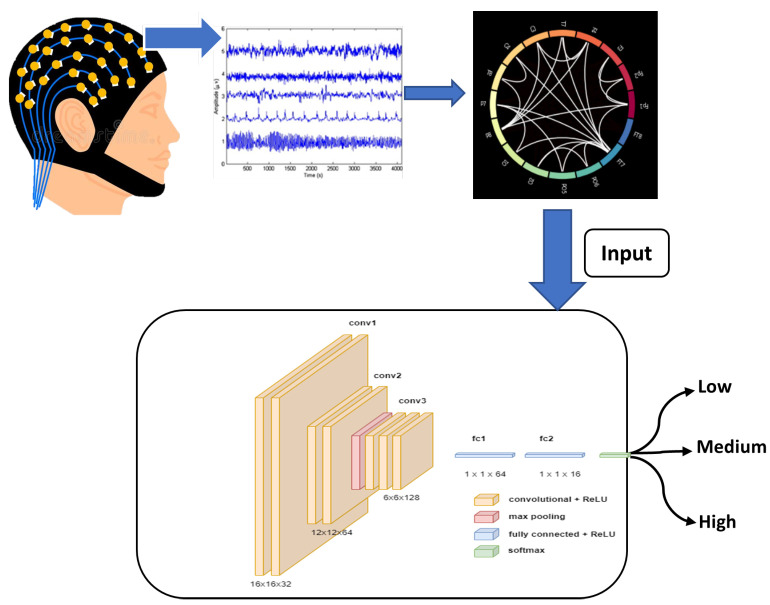
Overview of the classification workflow using EEG signals.

**Figure 3:**
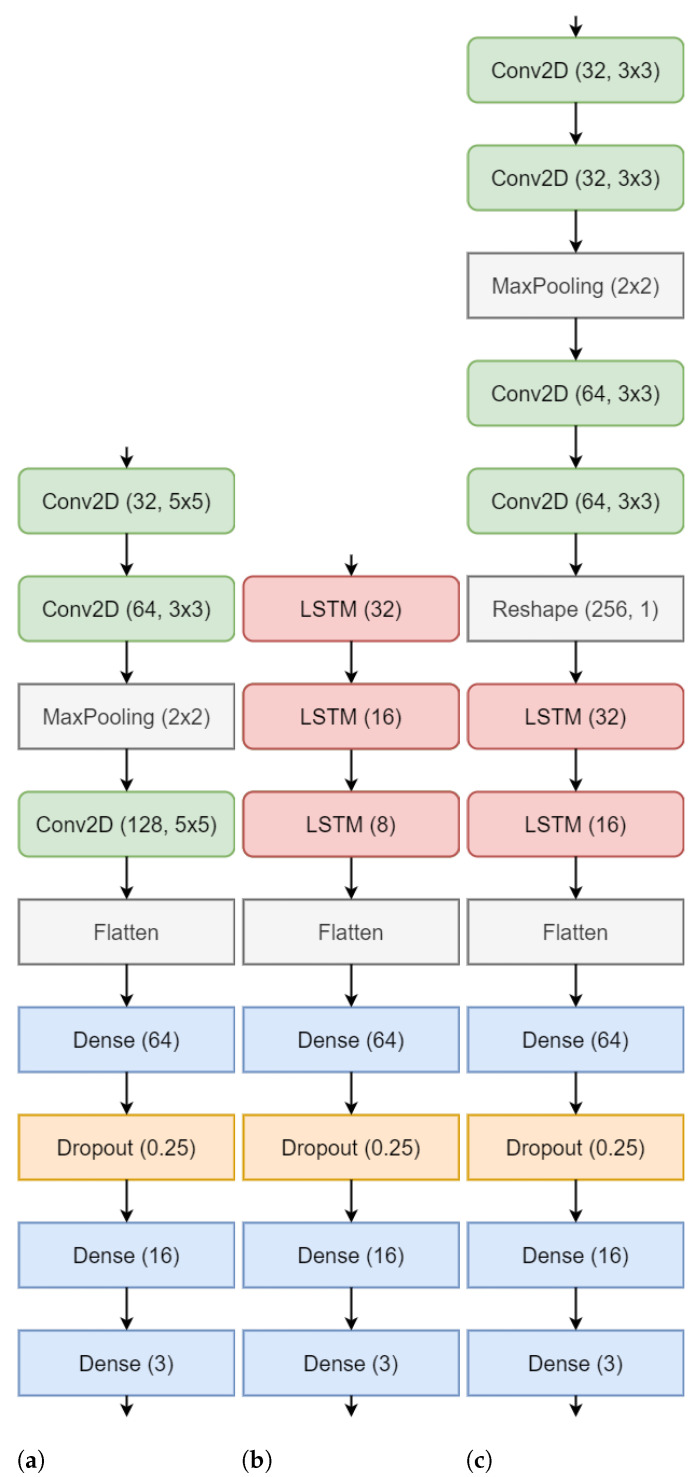
Model architectures for (**a**) CNN C-A (**b**) LSTM L-A (**c**) Conv-LSTM CL-A.

The classification of workload is implemented using three different variants of convolution and recurrent neural networks that provide different feature extraction and learning capabilities and a comparison of the performance is presented. The input to all the three networks were the connectivity matrices MI, PLV and PTE as described above. The shape of each of the matrix was 16×16. The networks were trained using Python 3.9 and Tensorflow 2.4 on Nvidia DGX server at Indian Institute of Technology, Roorkee. For processing the input and feeding it to the model, we used Tensorflow Datasets API and used 70,15,15 split for training, validation and testing data. As mentioned earlier, the n-back task was composed of 339 sessions, hence, we calculated a matrix corresponding to each session giving rise to 339 matrices for each participant. With the split of 70-15-15, there were 237, 51 and 51 matrices for training, validation and testing, respectively, for each of the 19 subjects. We used a batch size of 64 trained each model for 1000 epochs. During the training, early stopping [56] and learning rate scheduler [57] were used to improve the convergence time. The motivation and details of the networks used are as follows; The CNN classifier [58] was chosen based on the similarity that the input (which is a weighted square adjacency matrix) has to an image, as it’s ability to extract spatial features is superior unlike the primitive ANNs. We used a Regular CNN (Table 1) (consisting of the usual 2D convolution, pooling and batchnorm layers). For all the convolution layers of the models, stride of 1, ‘same’ padding, and ReLU [59] as activation was used. The last dense layer consisted of 3 units and softmax activation [60] for classifying the three levels of workload. Similarly, in LSTM (Table 2), the input was flattened and all LSTM layers make use of ReLU activation. In Conv-LSTM (Table 3), all Conv2D layers have ReLU activation. After reshaping the output, they are followed by LSTM layers, followed by 2 dense layers and a softmax layer same as the above models. The overview of the classification framework can be visualized as shown in Figure 2. Additionally, Figure 3 shows the architecture of the CNN, LSTM and the Conv-LSTM models used.

## 3. Results and Discussion

In this research, the efficacy of three different functional brain connectivity analysis methods (MI, PLV and PTE) to classify cognitive workload into high, medium and low using three different deep learning architectures (CNN, LSTM and Conv-LSTM) was investigated. Nineteen participants executed the the modern version of the n-back task on a computer screen with three levels of cognitive workload, high, medium and low.

**Figure 4:**
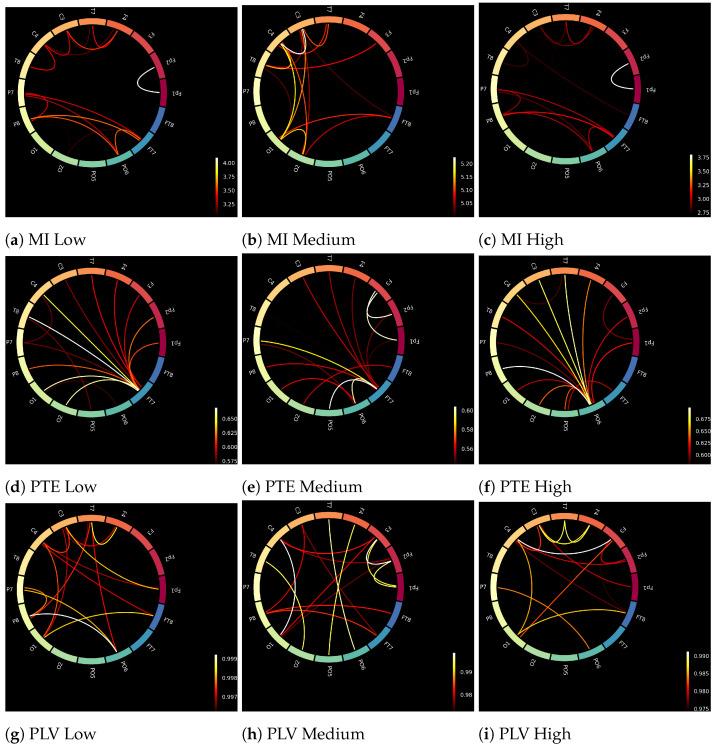
Brain connectivity maps of a random subject obtained through MI, PTE, and PLV for different workload states (low, medium, and high) using Brodmann atlas [61].

The input to the deep learning networks was 16 × 16 connectivity metrics. Sixteen brain regions were chosen from the brodmann atlas [61] to cover the different brain regions and at the same time keep the computations as fast as possible. Figure 4 shows the differences (for a random participant) between low, medium and high workloads of MI, PTE and PLV, respectively. Although the differences among the three connectivity metrics are visible, there are no explicit and visible differences among the three workload conditions, i.e., low, medium and high.

However, in the statistical analysis, significant differences were found among the three conditions. The mean accuracy (in percentage) for the three n-back condition was- 75.42 (SD = 16.10), 62.27 (SD = 15.64), 37.84 (SD = 14.18) for 1-back, 2-back and 3-back, respectively. There were significant differences among the groups (*F* (2, 75) = 40.22, *p* < 0.01, $\eta^{2}$ = 0.56). Similarly we found significant differences in the reaction time as well (1-back = 492.58 (SD = 91.1), 2-back = 673.58 (SD = 150.57), 3-back = 824.84 (SD = 147.32), ANOVA = *F* (2, 75) = 40.98, *p* < 0.01, $\eta^{2}$ = 0.48). Differences between all possible combinations (1 vs. 2, 1 vs. 3, 2 vs. 3) across both mean accuracy (in percentage) and mean reaction time (in ms) were also found to be significant (*p* < 0.01).

Based on the statistical results, we hypothesized that there will be differences in the brain connectivity matrices (although not visible to the naked eye) in the three workload settings and the deep learning classifiers will be able to utilize these differences for successful classification. It was expected that PTE would perform best in terms of connectivity metric, with it being directed and phase-specific.

**Figure 5:**
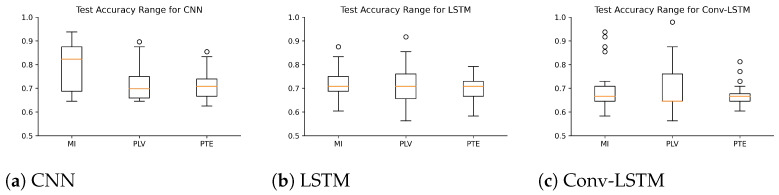
Box Plots representing the range of accuracy (with standard error) achieved by different subjects with deep learning architectures used (**a**) CNN (**b**) LSTM and (**c**) Conv-LSTM.

**Figure 6:**
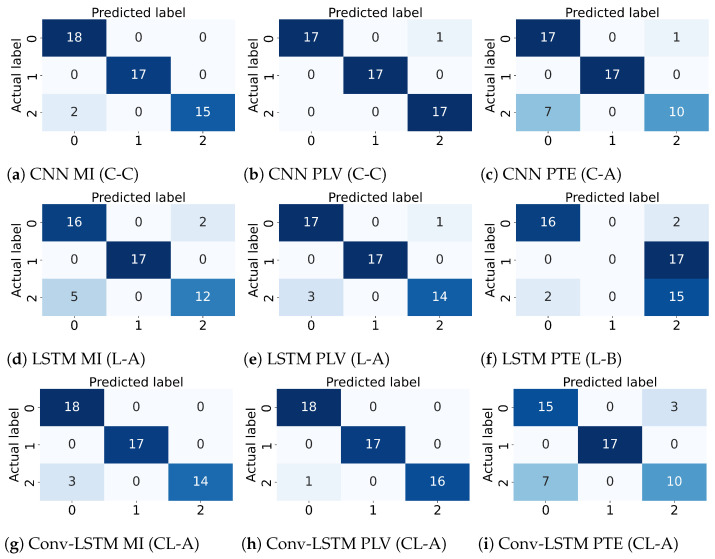
Confusion Matrix for the best performing subject for different combinations of the deep learning architectures (CNN, LSTM, and Conv-LSTM) and the functional connectivity metrics (MI, PLV and PTE).

**Figure 7:**
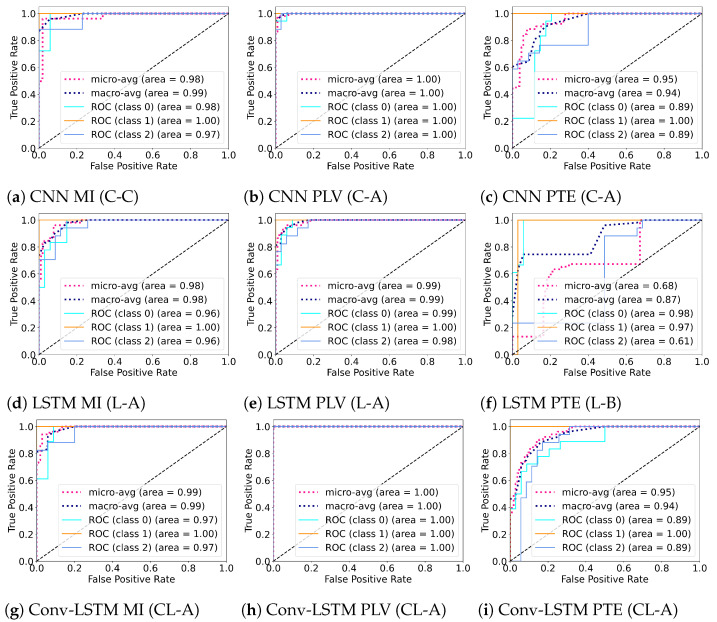
ROC (Receiver Operating Characteristics) curves for the best performing subject for different combinations of the deep learning architectures (CNN, LSTM, and Conv-LSTM) and functional connectivity metrics (MI, PLV and PTE).

Several experiments (ablation study) were performed to find best hyperparameter settings for the three deep learning architectures. The results of the ablation study are compiled in Table 4. As shown in Table 4, for MI, a mean accuracy of 80.87% was achieved with CNN, 71.87% was achieved with LSTM and 71.16% was achieved with Conv-LSTM. Similarly, for PLV a mean accuracy of 75.88% was achieved with CNN, 71.82% was achieved with LSTM and 69.68% was achieved with Conv-LSTM. Lastly, for PTE a mean accuracy of 71.16% was achieved with CNN, 69.63% was achieved with LSTM and 69.74% was achieved with Conv-LSTM. The highest accuracy (among all subjects) was achieved with the combination of PLV with Conv-LSTM and CNN at 97.92%. This is followed by MI with CNN at 95.83%. Besides the accuracy, Precision, Recall and F1-score of the classifiers are also reported in Table 5. Figure 5 shows the box-plot containing the accuracy and statistical results (standard error, quartiles, and outliers) of all the classifiers in combination with different functional connectivity methods. The combination of CNN and MI indicates the best classification performance. The achieved accuracy outperforms the state-of-the-art in multi-class classification in the context of workload classification in the n-back task with various EEG features and machine-learning algorithms. The comparison of the proposed method with others is given in Table 6. Since, the number of trials for the three workload settings were balanced, accuracy was indicative of the performance of the classifiers. Nevertheless, we reinforced the results with the analysis of the confusion matrices and ROC curves. Figure 6 shows the confusion matrix and Figure 7 shows the ROC curves for all combinations of the classifiers and the connectivity metrics of the best subject. From these figures, it can be substantiated that the classification performance of the models is high for the multiclass-classification problem as the true positive rate is high. The high value class-wise area under the curve shows that the classifier is able to learn and classify each class separately with high accuracy.

**Figure 8:**
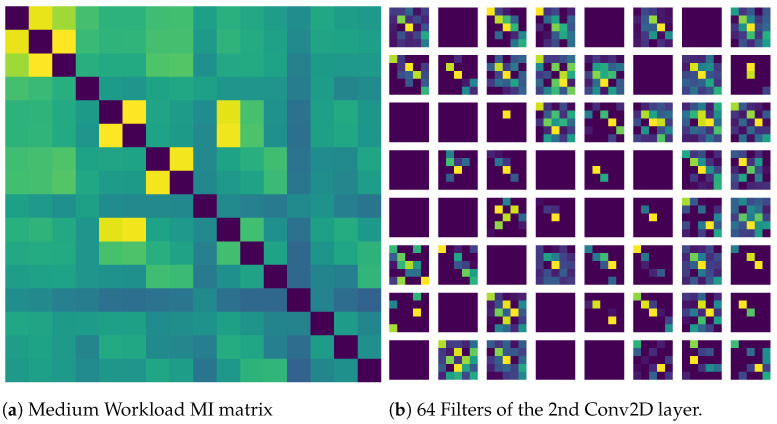
(**a**) Input given to the CNN network (**b**) Visualization of feature maps of the convolution layer in the CNN network.

Figure 8 shows the features learned by the CNN when MI was given as an input. MI was chosen as it gave the highest accuracy and similarly, input image of medium workload was chosen since the recall of medium workload was highest. It is visible that the filters are actually learning similar activation as in the input image indicating that the classifier was successful. Overall, given the consistent performance of the classifiers across all the metrics and the significant differences found in the statistical tests, it can be concluded that the classifier was successful.

Although state-of-the-art results were obtained, the study had some limitations. One important limitation of the study is the hypothesis itself. We hypothesized that there will be differences in the connectivity matrices in the three workload conditions. However, the study was limited to calculating the connectivity using raw(cleaned) EEG data. This was done to test whether all inclusive connectivity (not band limited) would yield conceivable differentiation in workload or not. This would have implications in making the entire framework close to real-time since band-limiting the signals would have increased the computational complexity. In the future we will consider doing a comparison with our approach and investigations in connectivity with different frequency bands to make a comprehensive and exhaustive hypothesis. Another limitation was the subject-dependent classification. The subject-dependent classifiers can extract subject-dependent features and can effectively tackle the issue of accuracy and generalization encountered in subject-independent EEG classifiers. However, it also gives rise to the issues of long collaboration sessions and collection of large quantities of data [38,39]. Lastly, the choice of 16 brain regions for computing the connectivity matrices. The choice of the brain regions could have been empirical instead of hypothesis and use-case driven. Exhaustive search and feature selection algorithms could be used in the future for validating the selection of brain regions empirically.

## 4. Conclusions

Workload Classification can be used as an indicator of the Emotional Intelligence and stability. The aim of the study was to build a fast and accurate workload classifier which can be extended to real-time workload classification. Real-time workload classification is an important and very useful cognitive construct for the development of robust BCI systems [62] and useful in several other domains like Virtual Reality [63] and Human-Machine Teaming [64]. In this research, EEG was chosen as the neuroimaging modality with its advantages of being cheap, portable and having high time resolution [65]. Model-free functional connectivity was chosen for the feature extraction with the concomitant advantages of being fast and associated with cognitive control in the context of mental workload [66]. Also, it has been shown that there are subject-specific differences in EEG-based functional connectivity measures [37].

Thereby, a combination of various directed/non-directed model-free brain functional connectivity algorithms and state-of-the-art deep learning algorithms were utilized for efficient subject-specific classification of cognitive workload into three levels, high, medium and low. Three functional brain connectivity algorithms (Mutual Information, Phase Transfer Entropy and Phase Locking Value) were used to generate the functional connectivity networks, which represents the neuronal interactions between the different regions of the brain. These connectivity networks are used as inputs to the classification models to classify different levels of workload. We employed three different deep learning architectures (CNN, LSTM and Conv-LSTM) for classification of cognitive workload. Intra-subject method of classification was applied on the data of 19 participants. The best classification performance was obtained with CNN in combination of each of the three connectivity networks over LSTM and Conv-LSTM. CNN outperforms the other two deep learning architectures because of the spatial information provided by the connectivity analysis in the form of input data upon which the classification is being performed. With CNN, MI produces the best classification results with an accuracy of 80.87%, followed by CNN with PLV with an accuracy of 75.88% and LSTM with MI with an accuracy of 71.87%.

We achieved state-of-the-art accuracy for multi-class workload classification using EEG and functional connectivity. From the results, it can be concluded that indeed EEG-based model-free functional connectivity metrics, when combined with deep-learning, provides an accurate, reliable and fast method of classifying cognitive workload. Although there is not much literature available on this, it was hypothesized that the connectivity method PTE will outperform MI and PLV as PTE is the only connectivity measure that is phase-specific and directed in nature. However, in our experiments MI outperformed PTE in the classification performance. This can be due to the fact that this study had lesser number of participants’ and the choice of brain regions. Therefore, no significant conclusions can be made about which model-free connectivity measure is the best. A future study can be performed with higher number of participants and different permutations and combinations of brain regions to make better and clear conclusions regarding the comparative analysis of the different connectivity measures.

Since these brain connectivity methods enable extremely rapid (specially MI) and accurate connectivity matrix generation from raw EEG data, the proposed architecture (a combination of MI/PLV/PTE and state-of-the-art CNN) can be used for effective and efficient cognitive state monitoring and other BCI applications. In addition to that, brain connectivity coupled with hybrid deep learning architectures can be used to classify higher-order cognitive processes like executive functioning and complex decision-making in the future. The subject-specific classification also sanctions the analysis and extraction of subject-specific features. Together, this could enable BCIs to become more reliable and efficient exponents of effective state monitoring in complex real world scenarios.

## Figures and Tables

**Table 1:** Configuration of CNN Architectures used for the ablation study. C-A, C-B and C-C refers to the three variations of CNN Networks. The bottom half of the table is common to all the three variations.

C-A	C-B	C-C
Input [16, 16, 1]	Input [16, 16, 1]	Input [16, 16, 1]
Conv2D (32, 5×5)	Conv2D (32,5×5)	Conv2D (32, 5×5)
Conv2D (64, 3×3)	Conv2D (64,3×3)	Conv2D (64, 5×5)
MaxPooling (2×2)	MaxPooling (2×2)	MaxPooling (2×2)
Conv2D (128, 5×5)	Conv2D (128, 5×5)	Conv2D (128, 3×3)
	Conv2D (128, 5×5)	
Flatten		
Dense (64)		
Dropout (0.25)		
Dense (16)		
Dense (3)		

**Table 2:** Configurations of LSTM Architectures used for the ablation study. L-A, L-B and L-C refers to the three variations of LSTM Networks. The bottom half of the table is common to all three variations.

L-A	L-B	L-C
Input [256, 1]	Input [256, 1]	Input [256, 1]
	LSTM (64)	LSTM (64)
LSTM (32)	LSTM (32)	LSTM (32)
LSTM (16)	LSTM (16)	LSTM (16)
LSTM (8)	LSTM (8)	LSTM (16)
Flatten		
Dense (64)		
Dropout (0.25)		
Dense (16)		
Dense (3)		

**Table 3:** Configuration of Conv-LSTM Architectures used for the ablation study. CL-A, CL-B and CL-C refers to the three variations of Conv-LSTM Networks. The bottom half of the table is common to all the three variations.

CL-A	CL-B	CL-C
Input [16, 16, 1]	Input [16, 16, 1]	Input [16, 16, 1]
Conv2D (32, 3×3)	Conv2D (16, 3×3)	Conv2D (32, 3×3)
Conv2D (32, 3×3)	Conv2D (16, 3×3)	Conv2D (32, 3×3)
MaxPooling (2×2)	MaxPooling (2×2)	MaxPooling (2×2)
Conv2D (64, 3×3)	Conv2D (64, 3×3)	Conv2D (64, 3×3)
Conv2D (64, 3×3)	Conv2D (64, 3×3)	Conv2D (64, 3×3)
Reshape (256, 1)	Reshape (256, 1)	Reshape (256, 1)
LSTM (32)	LSTM (64)	LSTM (64)
LSTM (16)	LSTM (16)	LSTM (32)
		LSTM (16)
Flatten		
Dense (64)		
Dropout (0.25)		
Dense (16)		
Dense (3)		

**Table 4:** Ablation Study of different variations of the hyper-parameter combinations for used classifiers as described in Table 1, Table 2 and Table 3.

Methods	Best Subject			Average Accuracy ± Std. Dev.		
	MI	PLV	PTE	MI	PLV	PTE
**CNN**						
C-A	93.75	89.58	85.42	80.87 ± 10.24	74.07 ± 08.28	71.16 ± 06.38
C-B	91.67	89.58	83.33	80.87 ± 10.29	71.49 ± 10.85	71.05 ± 10.85
C-C	95.83	97.92	79.17	80.21 ± 11.26	75.88 ± 11.01	70.72 ± 05.34
**LSTM**						
L-A	87.50	91.67	79.17	71.87 ± 06.56	71.82 ± 08.15	69.63 ± 05.66
L-B	85.42	79.17	81.25	69.52 ± 07.77	65.24 ± 07.79	67.00 ± 08.47
L-C	87.50	89.58	79.17	70.29 ± 07.30	69.41 ± 08.30	67.76 ± 06.80
**Conv-LSTM**						
CL-A	93.75	97.92	81.25	71.16 ± 10.03	69.68 ± 10.46	67.32 ± 05.05
CL-B	87.50	87.50	79.17	70.61 ± 08.27	68.64 ± 07.23	68.09 ± 04.73
CL-C	91.67	89.58	79.17	67.49 ± 07.12	67.87 ± 07.50	69.74 ± 05.54

**Table 5:** Precision, recall and F1-score for the different architectures used in the ablation study as described in Table 1, Table 2 and Table 3.

Methods	Precision			Recall			F1-Score		
	MI	PLV	PTE	MI	PLV	PTE	MI	PLV	PTE
**CNN**									
C-A	94.31	88.79	86.93	94.23	88.46	84.62	94.22	88.44	84.07
C-B	92.39	89.74	81.44	92.31	88.46	80.77	92.19	88.35	80.45
C-C	96.54	98.18	79.33	96.15	98.08	78.85	96.13	98.08	78.74
**LSTM**									
L-A	87.09	92.63	77.35	86.54	92.31	76.92	86.40	92.27	76.54
L-B	84.51	80.33	80.00	84.62	80.33	83.00	84.36	80.33	83.00
L-C	91.35	90.33	80.00	88.46	90.33	78.66	88.05	90.33	78.33
**Conv-LSTM**									
CL-A	95.05	98.18	81.44	94.23	98.08	80.77	94.17	98.07	80.45
CL-B	88.46	87.21	80.12	88.46	86.54	78.85	88.46	86.47	78.50
CL-C	90.48	90.44	78.85	90.38	90.38	78.85	90.38	90.26	78.81

**Table 6:** Comparison of the proposed work with state-of-the-art results. The comparison includes different features and classifiers used for EEG-based cognitive workload classification in the n-back task. The proposed work achieves the highest accuracy in multi-class classification.

Paper	Feature	Classifier	Accuracy	Subject Dependency	Number of Classes
Appriou et al. [24]	Preprocessed EEG	CNN	72.7%	Subject Specific	2 Classes
			63.7%	Subject Independent	
Dimitrakopoulous et al. [31]	Functional Connectivity (Pearson Correlation)	SVM classifier (RBF kernel and Least Squares Learning Method)	88 %	Subject Independent	2 Classes
Zhang et al. [25]	Topographic Maps	RNN and 3D CNN structures (R3DCNN)	88.9 %	Subject Independent	2 Classes
Zhang et al. [26]	Topographic Maps	Modified CNN	91.9%	Subject Specific	3 Classes
**Proposed**	Functional Connectivity (PLV)	Conv-LSTM, CNN	97.92%	Subject Specific	3 Classses

## Data Availability

The raw data will be made available on request by the authors, without undue reservation.
